# Improvement in bladder dysfunction after bladder transplantation of amniotic fluid stem cells in diabetic rats

**DOI:** 10.1038/s41598-018-20512-z

**Published:** 2018-02-01

**Authors:** Ching-Chung Liang, Sheng-Wen Steven Shaw, Yung-Hsin Huang, Yi-Hao Lin, Tsong-Hai Lee

**Affiliations:** 1 Female Urology Section, Department of Obstetrics and Gynecology, Chang Gung Memorial Hospital Linkou Medical Center, Taoyuan, Taiwan; 2 College of Medicine, Chang Gung University, Taoyuan, Taiwan; 3 Division of Obstetrics, Department of Obstetrics and Gynecology, Chang Gung Memorial Hospital Linkou Medical Center, Taoyuan, Taiwan; 4 Prenatal Cell and Gene Therapy Group, Institute for Women’s Health, University College London, London, UK; 5 Stroke Center and Department of Neurology, Chang Gung Memorial Hospital Linkou Medical Center, Taoyuan, Taiwan

## Abstract

To examine the effects of human amniotic fluid stem cells (hAFSCs) transplantation on bladder function and molecular changes in diabetic rats, 60 female Sprague-Dawley rats were used for study. Three groups were assigned including sham control rats, streptozotocin (STZ, 60 mg/kg)-induced diabetic rats and STZ-induced diabetic rats plus bladder hAFSCs transplantation. Compared to controls, diabetic rats had decreased body weight but increased bladder weight. Cystometries showed non-voiding contraction, residual volume, voided volume and intercontraction interval increased significantly in diabetic rats at week 4 and 12 after DM induction, but improved after hAFSCs transplantation. The immunoreactivities and mRNAs of nerve growth factor (NGF) decreased significantly in diabetic bladder at week 4 and 12 after DM induction, but recovered after hAFSCs transplantation. The immunoreactivities and mRNAs of M2 and M3 muscarinic receptor increased significantly in diabetic bladder at week 4 after DM induction but recovered after hAFSCs transplantation. The immunoreactivity of 8-hydroxy-20-deoxyguanosine increased significantly in diabetic bladder at week 4 and 12 after DM induction but reduced after hAFSCs transplantation. The present study showed bladder dysfunction in STZ-induced diabetic rats could be improved by hAFSCs transplantation into bladder, which may be related to the recovery of bladder NGF and muscarinic receptors.

## Introduction

Diabetes mellitus (DM) is a metabolic disease with multiple serious complications including retinopathy, neuropathy and nephropathy^1,2^. Lower urinary tract symptoms (LUTS) are found in more than 80% of diabetic patients such as urine retention and incontinence that are usually resistant to currently available therapies^3,4^. The precise mechanism responsible for DM-related LUTS has not been completely clarified, but previous studies suggested that alterations in the bladder smooth muscle, neuronal degeneration and urothelial dysfunction could be the cause of DM-related bladder dysfunction^4,5^. Oxidative stress has also been reported to be associated with the development of diabetic cystopathy in streptozotocin-induced diabetic rats^6,7^. However, the role of oxidative stress in diabetic bladder dysfunction remains to be clarified.

In recent years, cellular therapy has been conducted for bladder dysfunction, but only few studies have been reported such as the use of adipose tissue-derived stem cells to improve diabetic bladder dysfunction in streptozotocin (STZ) induced diabetic rats^8,9^. However, adult stem cells from adipose tissue have the disadvantages of restricted differentiation potential and a shorter life span compared to amniotic fluid stem cells^10^. On the other hand, mesenchymal stem cells such as amniotic fluid stem cells have the advantage of immune-modulatory properties which make these cells a good candidate for xenotransplantation^11–13^. In this study, we investigated the effects of human amniotic fluid stem cells (hAFSCs) transplantation to improve bladder function and molecular changes using a STZ-induced diabetic rat model.

## Materials and Methods

### Animal model

All protocols were approved by the Institutional Ethics Committee for the Care and Use of Experimental Animals and the Institutional Review Board of Linkou Chang Gung Memorial Hospital. Efforts were made to minimize suffering, reduce the number of animals used, and utilize alternatives to *in vivo* techniques, if available. All procedures were performed in accordance with the National Institute of Health Guide for the Care and Use of Laboratory Animals (NIH Publications No. 80-23) revised in 1996.

**Figure 1:**
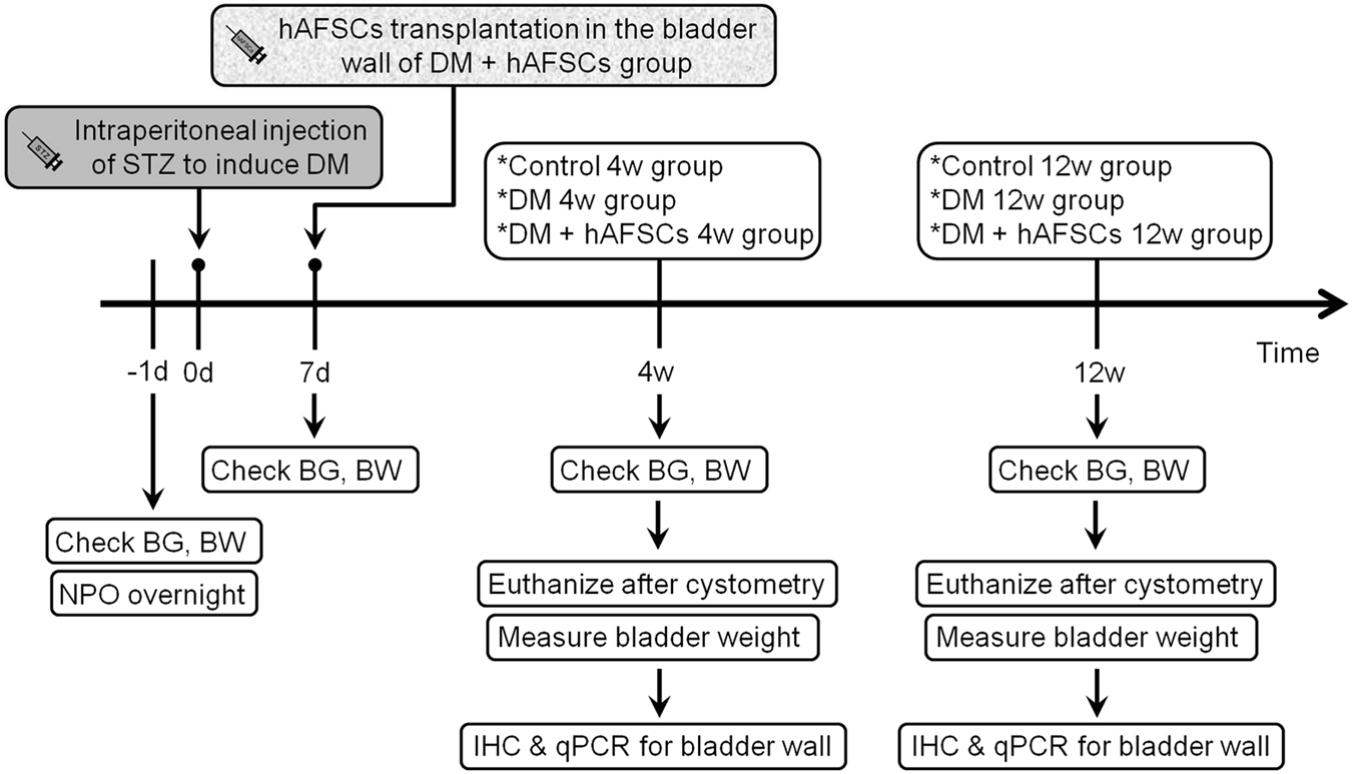
Schema of the experimental procedure. BS = blood sugar, BW = body weight, DM = diabetes mellitus, hAFSCs = human amniotic fluid stem cells, IHC = immunohistochemistry, NPO = nill per os, PCR = real-time polymerase chain reaction, STZ = streptozotocin.

Female Sprague-Dawley rats, aged 13–15 weeks and weighted 240–280 gm, were maintained at 21–23 °C room temperature and 47% humidity with a 12-hour light-dark cycle and free access to standard laboratory chow and tap water. Three groups were assigned: [1] sham control group with single intraperitoneal injection of 0.1 M citrate buffer solution; [2] diabetic rats induced by single intraperitoneal injection of streptozotocin (STZ, 60 mg/kg) diluted in 0.1 M citrate buffer solution after 24-h fasting according to the previous study^3^; [3] STZ-induced diabetic rats plus hAFSCs transplantation by using local injection of 3 × 10^6^ hAFSCs cells in 0.3 mL phosphate buffer solution (PBS) into bladder wall at day 7 after STZ injection. All rats received bladder function test using conscious cystometry at week 4 and 12 after sham treatment, STZ treatment or hAFSCs transplantation (n = 10 in each time point). Expressions of nerve growth factor (NGF), M2-muscarinic receptor (M2) and M3-muscarinic receptor (M3) were measured by immunohistochemistry and real-time polymerase chain reaction (PCR). Expressions of calcitonin gene-related peptide (CGRP), Substance P and 8-hydroxy-20-deoxyguanosine (8OHdG) were measured by immunohistochemistry. The experimental procedure is shown in Fig. 1.

### Induction of diabetes

Successful induction of DM was confirmed by measurement of serum glucose level at day 7 after STZ administration. Sham controls were treated with a similar volume of buffer instead of STZ. The rats were allowed to continue feeding on their respective diets until euthanasia. Blood glucose levels were measured with the ACCU-CHEK advantage blood glucose monitoring system (Roche Diagnostics, Indianapolis, IN). Routine test was carried out every week by taking a drop of blood from the rat tail vein to estimate the glucose level with BM-Test-Glycemie strips (Boehringer-Manheim, Lewes, East Sussex, UK). Animals with blood glucose 300 mg/dL or greater (fasted for 12 hours before measurement) were deemed diabetic and suitable for study.

### Isolation and characterization of hAFSC for transplantation

The hAFSCs were obtained from the freshly collected amniotic fluid by routine amniocentesis from healthy pregnant donors in 15–20 gestational weeks. Cells were cultured in StemPro® MSC serum free medium supplemented with 10% fetal bovine serum (Invitrogen, Carlsbad, CA) and incubated at 37 °C with 5% carbon dioxide. Culture medium was changed every 3–4 days. The specific surface antigens of hAFSCs were characterized by flow cytometry analyses as shown in our previous work^14^. The cultured cells were trypsinized and stained with phycoerythrin (PE)-conjugated antibodies against CD44, CD73, CD90, CD105, CD117 and CD45 (BD PharMingen, CA). Thereafter, the cells were analyzed using the Calibur flow cytometer (Becton Dickinson, Heidelberg, Germany). Passage 6–8 hAFSCs were collected and prepared to a final concentration of 3 × 10^6^ cells/0.3 mL PBS. In the hAFSCs-transplanted groups, 3 × 10^6^ collected hAFSCs were transplanted at day 7 after STZ induction by injection into the 5 sites of rat bladder wall (anterior, posterior, bilateral and dome) according to previous study^15^ under inhalation anesthesia with 3% isoflurane in a mixture of air with nitrous oxide and oxygen (70%/30%). During each local injection, the syringe was pushed backwards to confirm the needle was not present inside the vessel and was inside the muscular tissue of bladder. The treatment dose of hAFSCs was determined according to the previous study that used adipose tissue-derived stem cells to treat bladder dysfunction of STZ-diabetic rats^8^.

### Cystometric study

All rats received suprapubic tube implantation under 3% isoflurane inhalation 3 days prior to implementation of cystometry. The animals were placed in special metabolic cages (Med Associates, Saint Albans, VT) to perform conscious cystometric studies at week 4 and 12 after sham treatment, STZ treatment or hAFSCs transplantation according to our previous study^16^. All the cystometric parameters on 5 representative micturition cycles were collected for analysis. The following five cystometric variables^17^ were investigated including peak voiding pressure, non-voiding contraction, voided volume, intercontraction interval (ICI) and residual volume. The peak voiding pressure measured the peak of the detrusor contraction. The non-voiding contraction measured the number of undulating curves present in the filling phase. The voided volume per micturition was the volume expelled at micturition. The ICI between 2 consecutive contractions was calculated in each micturition cycle. The residual volume was the perfused volume subtracting the voided volume. Cystometry Analysis Version 1.05 (Catamount Research and Development) was used for cystometric analysis.

### Immunohistochemistry

Animals were euthanized after cystometry, and bladders were transected at the level of ureteral orifice. The dissected bladders were fixed in an optimal cutting temperature compound, frozen in powdered dry ice and stored at −80 °C. The bladders were then subjected to cryosection at 10 μm thickness under −18 °C.

Immunostaining against NGF, M2 and M3, CGRP, Substance P, 8OHdG in fresh-frozen bladder sections was performed with avidin-biotin peroxidase method. First, fresh-frozen sections were fixed for 10 minutes in acetone for NGF, CGRP, Substance P and 8OHdG, and 10 minutes in 4% paraformaldehyde for M2 and M3, air dried and then rinsed with PBS. After blocking with Dako REAL peroxidase blocking solution (code S2023, DAKO Corp, Carpinteria, CA) for 20 minutes, sections were washed and incubated for 18–20 hours at 4 °C with a rabbit polyclonal antibody directed against NGF (1:700, OriGene Technologies, Inc. Rockville, MD), M2 (1:700, Millipore, Temecula, CA) and M3 (1:200, Santa Cruz Biotechnology, Santa Cruz, CA), goat polyclonal antibody directed against CGRP (1:200, Abcam, Cambridge, MA, USA), mouse monoclonal antibody directed against Substance P (1:75, Abcam, Cambridge, MA, USA) and 8OHdG (1:100, Santa Cruz Biotechnology, Santa Cruz, CA). Then, sections were washed and incubated for one hour using biotinylated secondary antibodies at 1:500 dilution (Vector Laboratories, Burlingame, CA). Negative controls were performed without primary antibody and without primary and secondary antibodies (Supplemental Fig. 1. Negative controls). Staining was developed with 3,3′-diaminobenzidine plus hydrogen peroxide as the chromogen. Image-Pro Plus Software (Media Cybernetics, Silver Spring, MD) was used for immunoreactivity measurement. The ratio of the immunoreactivity level of STZ-diabetic rats with or without hAFSCs transplantation to that of control rats was determined for NGF, M2 and M3, CGRP, Substance P and 8OHdG.

### Real-time polymerase chain reaction

Real-time PCR was carried out according to the manufacturer’s protocol. Total RNAs were prepared using a Trizol reagent (Invitrogen, Carlsbad, CA) and incubated in reverse transcription mixture at 25 °C for 5 minutes, 50 °C for one hour, 70 °C for 15 minutes; finally, the tubes were cooled to 4 °C for 5 minutes. Gene expression for NGF, M2 and M3 in the bladder tissue was analyzed by real-time PCR using inventoried TaqMan assays from Applied Biosystems (Life Technologies, Grand Island, NY). The NGF, M2 and M3 assays codes were Rn01533872-m1, Rn02532311-s1 and Rn00560986-s1, respectively (Applied Biosystems, Oster City, CA). GAPDH assay codes (Rs99999916-s1) were used as an endogenous control to allow for quantification of relative gene expression. Thermal cycling and fluorescence detection were performed using an ABI Prism 7900HT Sequence Detection System (Applied Biosystems, Oster City, CA). PCR conditions were 50 °C for 2 minutes, 95 °C for 10 minutes, followed by 40 cycles at 95 °C for 15 seconds and 60 °C for one minute. The data were calculated using the 2[−Delta Delta C(T)] method^18^. A ratio of the mRNA level of STZ-diabetic rats with or without hAFSCs transplantation to that of control rats was determined. The values were summated and expressed as mean ± SD and were compared statistically among the three groups and among different time points in each group.

### Statistical analysis

The data were analyzed statistically using one-way analysis of variance test followed by Tukey test. Values were considered significant if *P* < 0.05. Prism 5 software for statistical analysis (GraphPad, San Diego, CA) was used for all analysis.

## Results

### Blood sugar, and body and bladder weight in diabetic rats improved after hAFSCs transplantation

In Table 1, the body weight of diabetic rats was significantly lower than that of controls at week 4 and 12 after DM induction. Bladder weight and blood sugar were increased significantly at week 4 and 12 in diabetic rats as compared to control group. The hAFSCs transplantation caused significant improvement of blood sugar level and bladder weight and increase of body weight in diabetic rats at week 12 after DM induction.

**Table 1:** Blood glucose and body and bladder weight in control, diabetic rats (DM) and diabetic rats with hAFSCs transplantation (DM + hAFSCs).

Group (N = 10) (Mean ± SD)	Body weight (gm; Initial)	Body weight (gm; Final)	Bladder weight (mg; Final)	Blood glucose (mg/dL; Initial)	Blood glucose (mg/dL; Final)
Control 4 w	264.9 ± 9.5	300.9 ± 9.5	151.3 ± 32.2	138.7 ± 13.1	136.3 ± 8.9
DM 4 w	257.1 ± 15.1	228.0 ± 9.0*	267.5 ± 52.0*	138.3 ± 10.0	426.1 ± 34.8*
DM + hAFSCs 4 w	260.7 ± 15.7	249.9 ± 28.1*	260.8 ± 22.5*	142.3 ± 15.9	410.1 ± 59.8*
*P value*	0.353	<0.0001	<0.0001	0.815	<0.0001
Control 12 w	260.3 ± 10.7	324.4 ± 22.1	166.5 ± 20.4	132.8 ± 12.9	144.3 ± 6.9
DM 12 w	244.8 ± 15.6	233.9 ± 20.3*	444.3 ± 80.4*	135.1 ± 18.8	458.1 ± 63.1*
DM + hAFSCs 12 w	250.5 ± 13.4	259.8 ± 28.7*	368.5 ± 55.2*†	139.4 ± 18.6	381.1 ± 41.4*,^‡^
*P value*	0.119	<0.0001	<0.0001	0.693	<0.0001

^†^*P* < 0.05 vs. DM (12 w).

^‡^*P* < 0.001 vs. DM (12 w).

^*^P < 0.0001 vs. control (4 w and 12 w).

N = 10 rats in each group.

Initial body weight: body weight measured before sham treatment, STZ treatment or hAFSCs transplantation.

Final body weight: body weight measured 4 w and 12 w after sham treatment, STZ treatment or hAFSCs transplantation.

Final bladder weight: bladder weight measured 4 w and 12 w after sham treatment, STZ treatment or hAFSCs transplantation.

### Bladder dysfunction subsequent to DM induction is improved after hAFSCs transplantation

**Figure 2:**
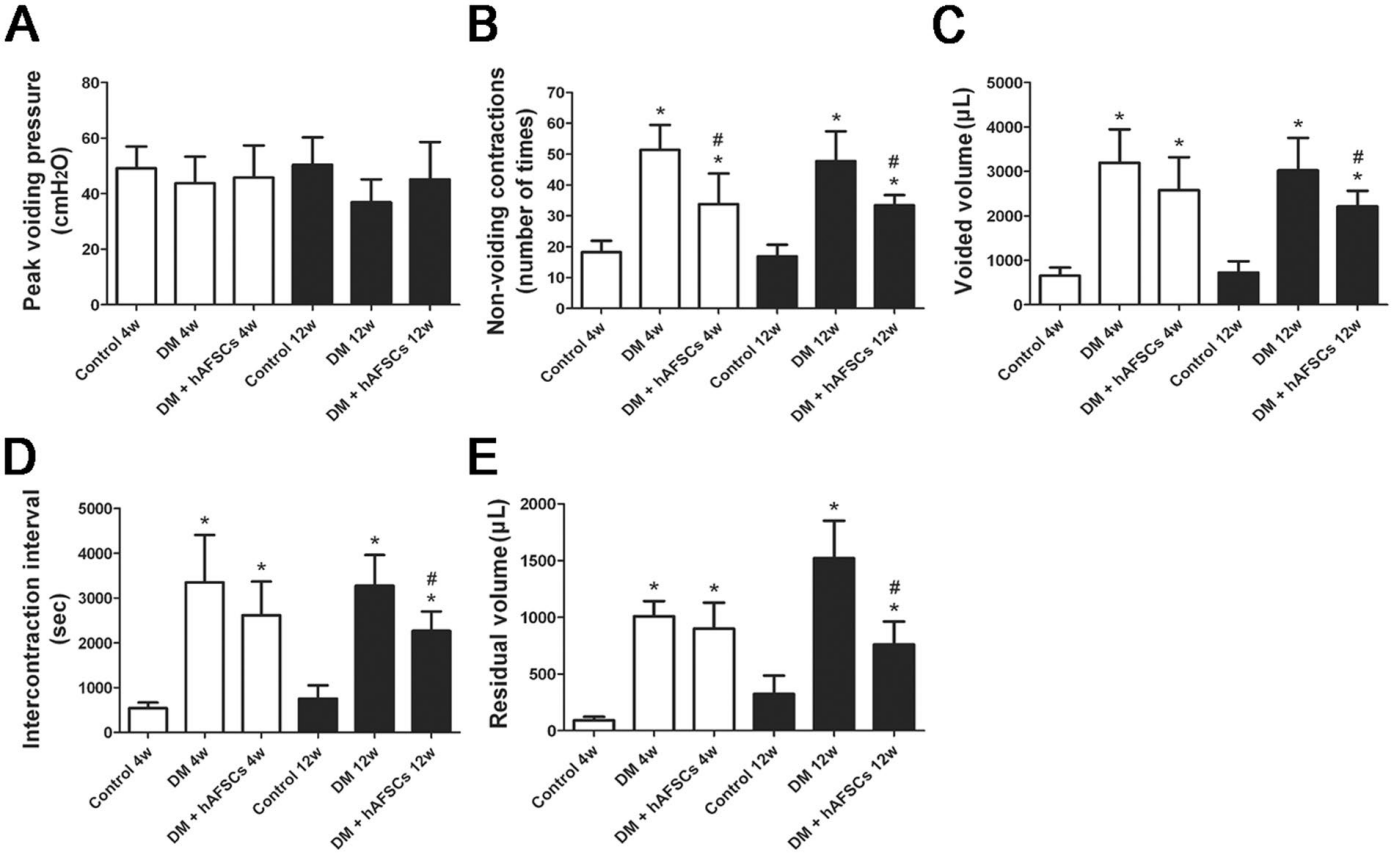
Cystometric results in the control, STZ-induced diabetic rats (DM) and diabetic rats + hAFSCs transplantation (DM + hAFSCs) are presented. Cystometric variables include peak voiding pressure (**A**), nonvoiding contractions (**B**), voided volume (**C**), intercontraction interval (**D**), and residual volume (**E**). Bladder dysfunction of the STZ-induced diabetic rats can be improved after hAFSCs transplantation. *P < 0.05 vs. control group. ^#^*P* < 0.05 vs. diabetic rat. N = 10 in each time point.

Cystometric analysis showed an increase of non-voiding contraction, voided volume, ICI and residual volume in diabetic rats at week 4 and 12 as compared to the control group (Fig. 2). However, there was no significant change in peak voiding pressure at week 4 and 12 after DM induction. The hAFSCs transplantation improved the non-voiding contraction, voided volume, ICI and residual volume at week 4 and 12 after DM induction.

### Expressions of NGF, CGRP, Substance P, M2 M3 and 8OHdG are improved after hAFSCs transplantation

**Figure 3:**
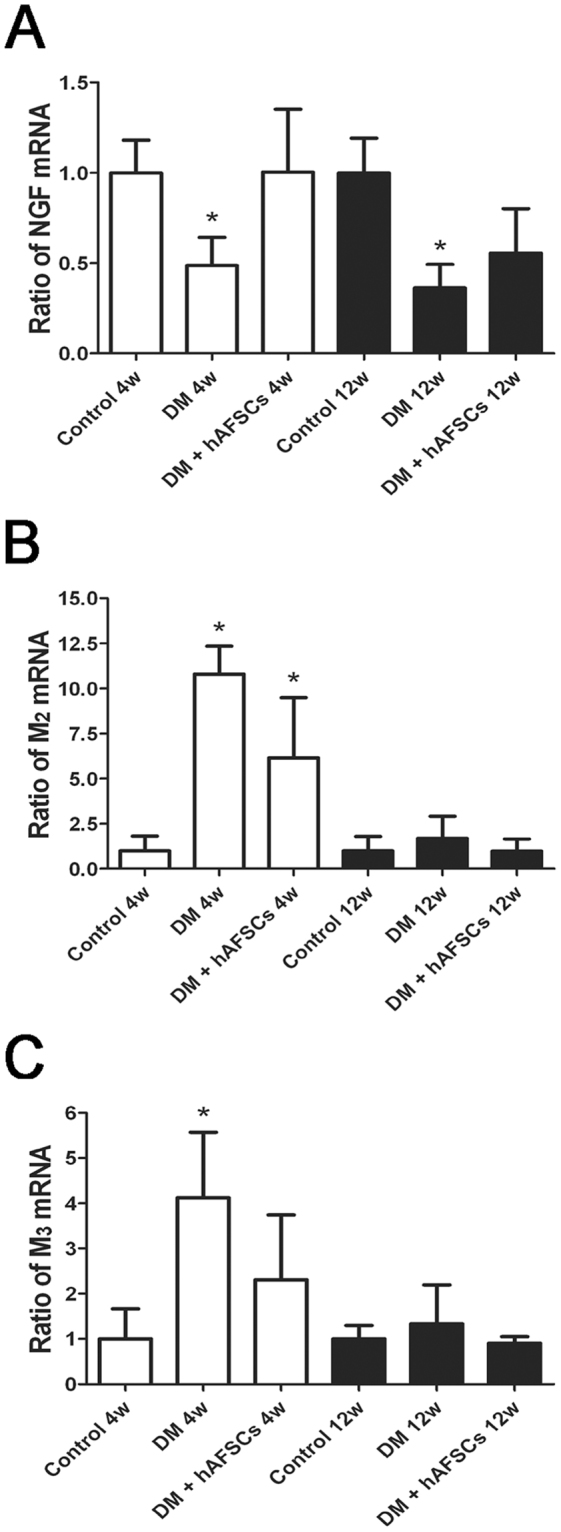
Relative mRNA expressions of nerve growth factor (NGF, **A**), M2-muscarinic receptor (M2, **B**) and M3-muscarinic receptor (M3, **C**) in the bladder of control, STZ-induced diabetic rats (DM) and diabetic rats + hAFSCs transplantation (DM + hAFSCs). Expressions of M2 and M3 mRNAs are increased, and expression of NGF mRNA is decreased in the diabetic rats at week 4 and 12 as compared to controls. After hAFSCs transplantation, expressions of NGF, M2 and M3 recover to near the control levels. Bar indicates 20 μm. *P < 0.05 vs. control group. N = 10 in each time point.

**Figure 4:**
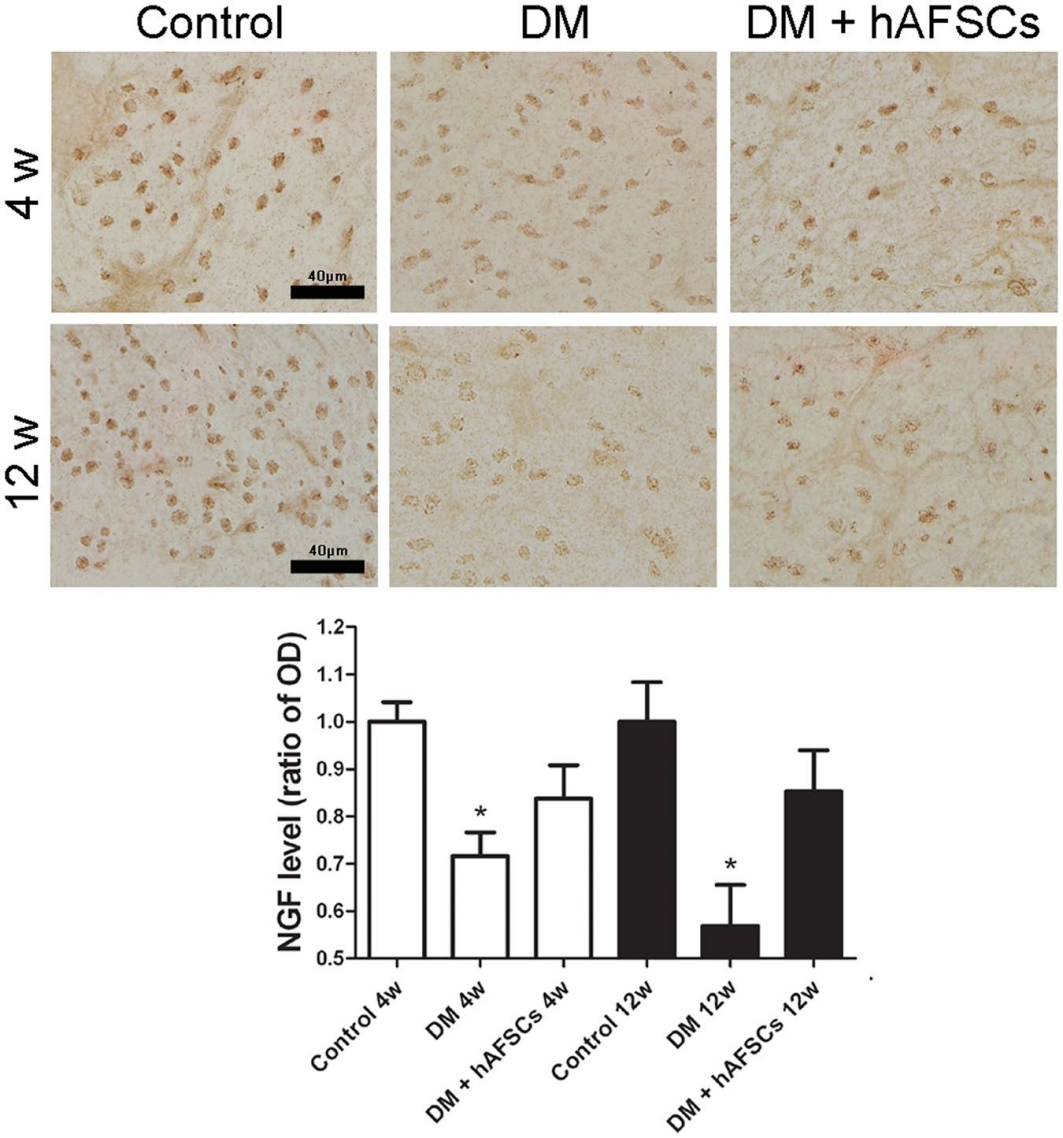
Temporal expressions of nerve growth factor (NGF) immunoreactivity in the bladder of control, STZ-induced diabetic rats (DM) and diabetic rats + hAFSCs transplantation (DM + hAFSCs). The immunoreactivity of NGF can be seen in control rats (**A**), diabetic rats (**B**), and diabetic rats + hAFSCs transplantation (**C**) at 4 weeks, and in control rats (**D**), diabetic rats (**E**), and diabetic rats + hAFSCs transplantation (**F**) at 12 weeks. When compared to the controls, the expressions of NGF in the diabetic rats are significantly decreased, but can be recovered to near the control level after hAFSCs transplantation. OD = optical density. Bar indicates 40 μm. *P < 0.05 vs. control group. N = 10 in each time point.

**Figure 5:**
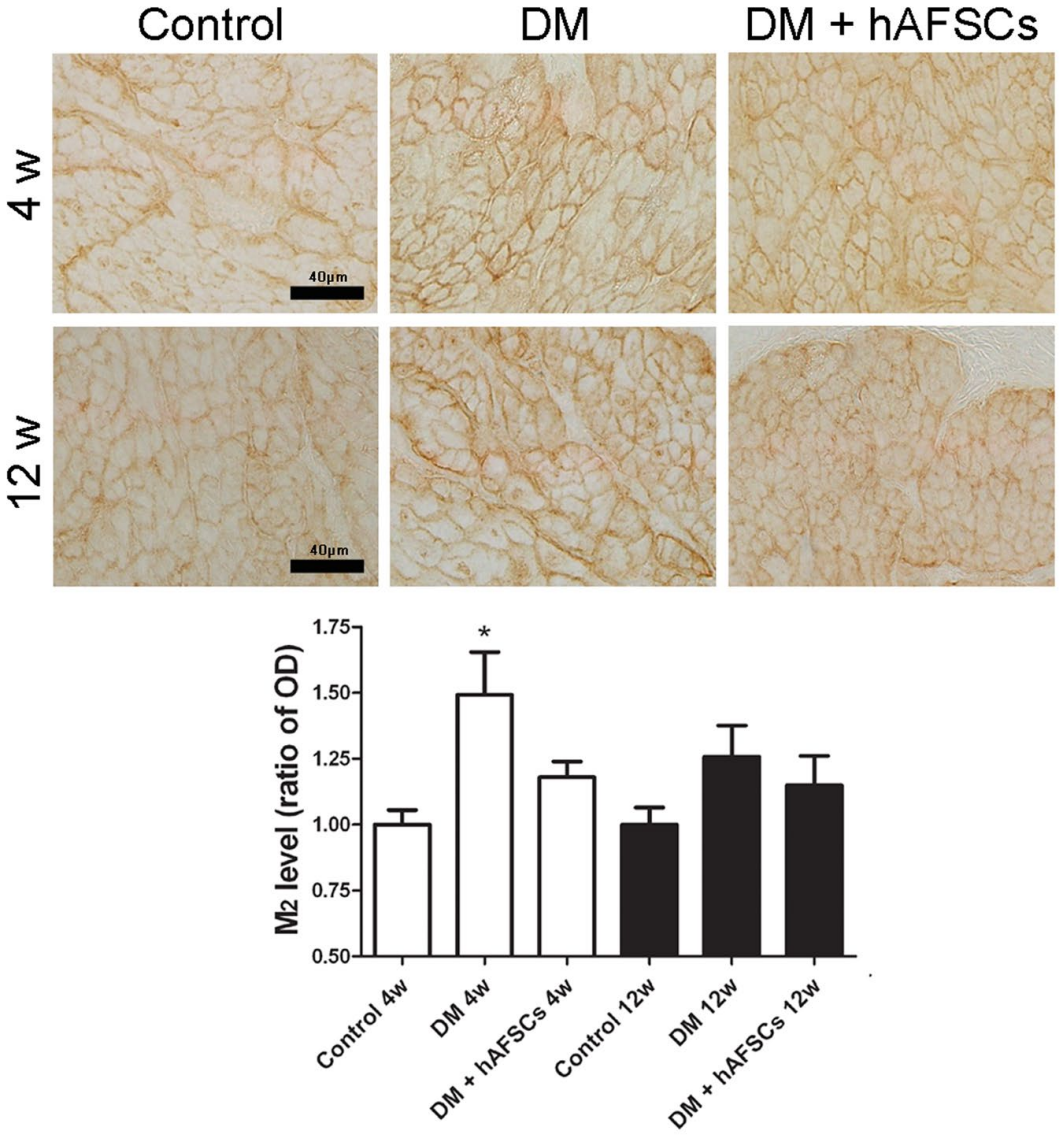
Temporal expressions of M2-muscarinic receptor (M2) immunoreactivity in the bladder of control, STZ-induced diabetic rats (DM) and diabetic rats + hAFSCs transplantation (DM + hAFSCs). The immunoreactivity of M2 can be seen in control rats (**A**), diabetic rats (**B**), and diabetic rats + hAFSCs transplantation (**C**) at 4 weeks, and in control rats (**D**), diabetic rats (**E**), and diabetic rats + hAFSCs transplantation (**F**) at 12 weeks. When compared to the controls, the expressions of M2 in the diabetic rats are increased, but can be recovered to near the control level after hAFSCs transplantation. OD = optical density. Bar indicates 40 μm. *P < 0.05 vs. control group. N = 10 in each time point.

**Figure 6:**
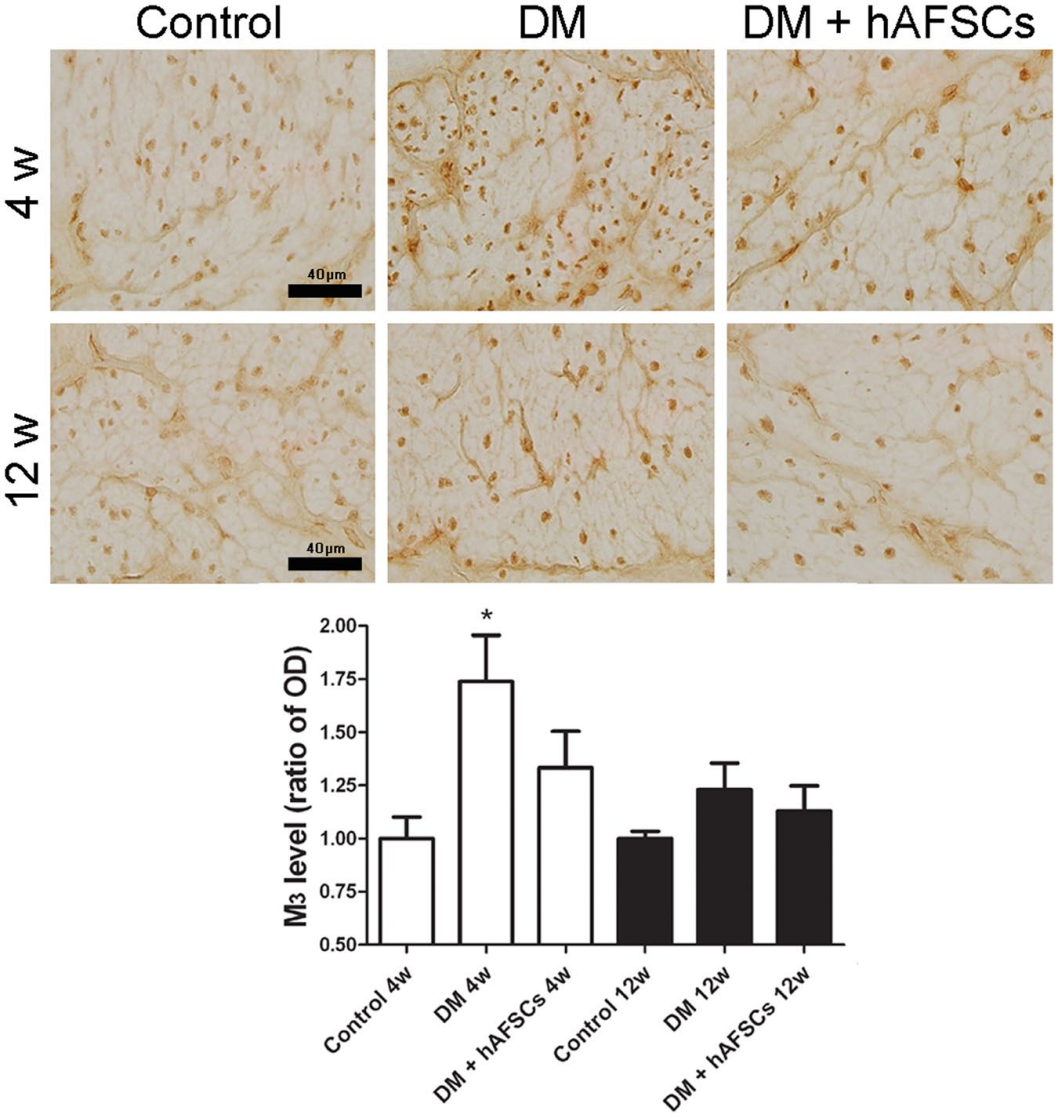
Temporal expressions of M3-muscarinic receptor (M3) immunoreactivity in the bladder of control, STZ-induced diabetic rats (DM) and diabetic rats + hAFSCs transplantation (DM + hAFSCs). The immunoreactivity of M3 can be seen in control rats (**A**), diabetic rats (**B**), and diabetic rats + hAFSCs transplantation (**C**) at 4 weeks, and in control rats (**D**), diabetic rats (**E**), and diabetic rats + hAFSCs transplantation (**F**) at 12 weeks. When compared to the controls, the expressions of M3 in the diabetic rats are increased, but can be recovered to near the control level after hAFSCs transplantation. OD = optical density. Bar indicates 40 μm. *P < 0.05 vs. control group. N = 10 in each time point.

**Figure 7:**
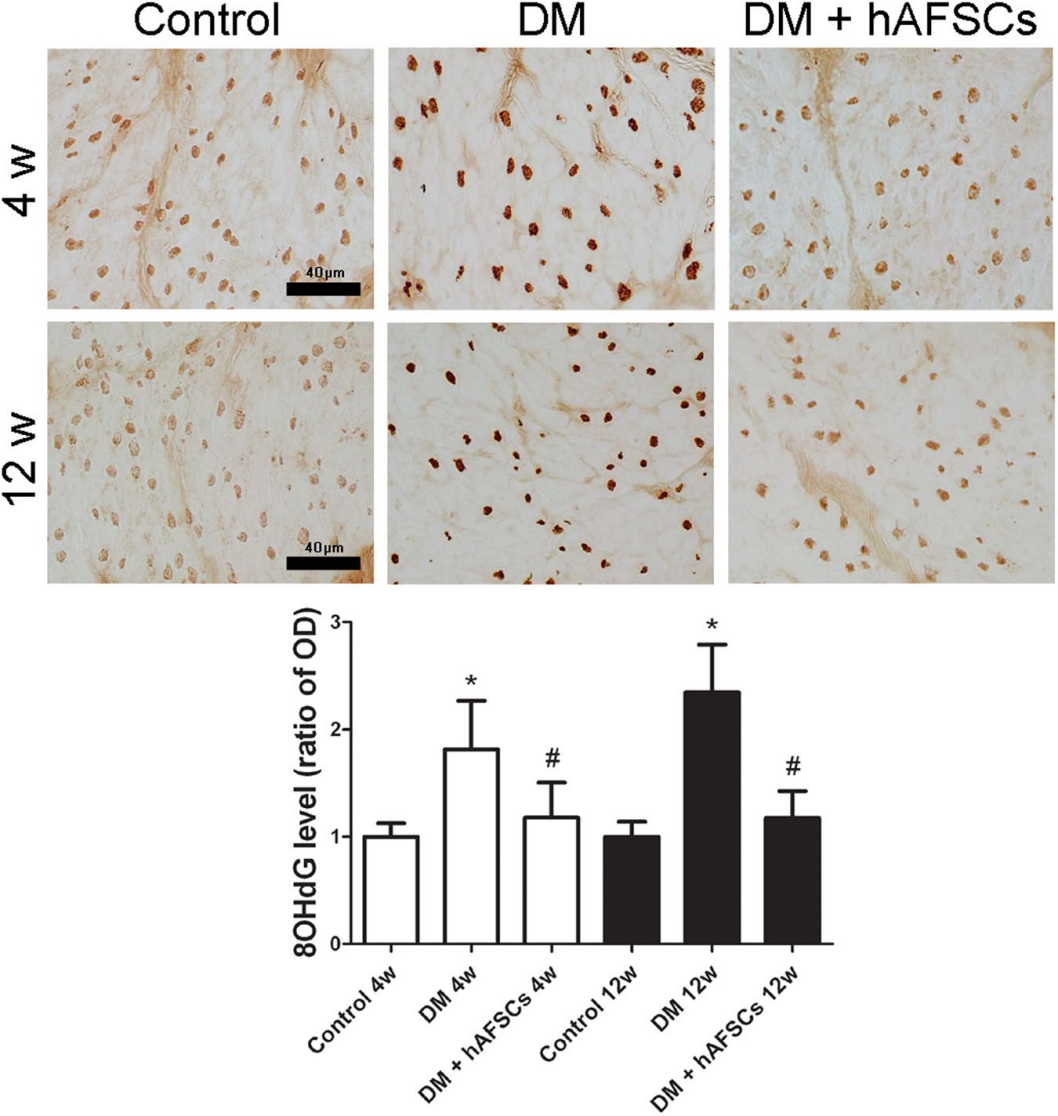
Temporal expressions of 8-hydroxy-20-deoxyguanosine (8OHdG) immunoreactivity in the bladder of control, STZ-induced diabetic rats (DM) and diabetic rats + hAFSCs transplantation (DM + hAFSCs). The immunoreactivity of 8OHdG can be seen in control rats (**A**), diabetic rats (**B**), and diabetic rats + hAFSCs transplantation (**C**) at 4 weeks, and in control rats (**D**), diabetic rats (**E**), and diabetic rats + hAFSCs transplantation (**F**) at 12 weeks. When compared to the controls, the expressions of 8OHdG in the diabetic bladder are increased at 4 and 12 weeks, but can be recovered to near the control level after hAFSCs transplantation. OD = optical density. Bar indicates 40 μm. *P < 0.05 vs. control group. ^#^*P* < 0.05 vs. diabetic rat. N = 10 in each time point.

The expressions of NGF immunoreactivities and mRNA decreased significantly in diabetic rats at week 4 and 12 after DM induction. The hAFSCs transplantation recovered the bladder NGF immunoreactivity and mRNA levels in diabetic rats to near the control levels (Figs 3 and 4). The immunoreactivities of sensory nerve marker, CGRP and Substance P, reduced in diabetic rats at week 4 and 12 after DM induction but increased after hAFSCs transplantation (Supplemental Fig. 2 for CGRP and Supplemental Fig. 3 for Substance P). The expressions of M2 and M3 immunoreactivities and mRNAs increased significantly in the bladder of diabetic rats at week 4 after DM induction as compared to the control rats (Figs 3, 5 and 6). However, the M2 and M3 immunoreactivitis and mRNAs recovered to near the control levels after hAFSCs transplantation at week 4 after DM induction. The 8OHdG immunoreactivity increased significantly in the diabetic bladder at week 4 and 12 after DM induction. After hAFSCs transplantation, the 8OHdG immunoreactivity reduced, but there was still significant difference between DM and DM + hAFSCs at week 4 and 12 (Fig. 7).

## Discussion

Bladder dysfunction in patients with DM can present with decreased voiding sensation, increased bladder capacity and poor emptying function^19^. Many of these voiding dysfunctions can be seen similarly in the STZ-induced diabetic rat^20,21^. Experimental studies demonstrated that the effects of STZ-induced diabetes on bladder function and bladder mass may occur at the onset of DM, aggravate to the maximal degree within 7 to 14 days, and then maintain relatively stable until 8 weeks^22,23^. At two months after STZ administration, decompensated bladder function can be seen in diabetic rats due to accumulation of oxidative stress products after prolonged hyperglycemia^4,24^. Daneshigari *et al*. observed a decrease in peak voiding pressure and an increase in residual urine in diabetic rats at 12 to 20 weeks after DM induction^24^. They advocated that diabetic bladders may undergo a transition from a compensated to a decompensated state in the STZ-induced diabetic rat at 9 to 12 weeks after STZ injection^24^.

Our results revealed that mean bladder weight and post-void residual urine of diabetic rats were greater than that in control rats at week 4 and 12 after STZ injection. Increased bladder weight and residual urine indicate the incomplete emptying in the hyposensitive diabetic bladder. However, we did not find a significant decrease in peak voiding pressure at week 12 after STZ administration. Sasaki *et al*. also reported no significant difference in maximal voiding pressure between diabetic and non-diabetic rats at week 12 after STZ administration^25^. In the present study, cystometry showed that mean voided volume and ICI increased significantly at week 4 and 12 after STZ administration. Previous studies noticed a similar result of time-dependent increase in ICI between week 4 and 12 after STZ administration^26^. However, Sasaki *et al*. found that ICI in the diabetic rats was significantly smaller than that in the control rats at week 4 and 12 after STZ administration^25^. The above results of ICI in the diabetic rats showed conflicting, and further study is indicated.

Diabetic bladder dysfunction may result from bladder nerve and smooth muscle dysfunction^4,5^. Hyposensitive distended bladder may result in an increase of ICI and residual urine, which may be compromised by nervous dysfunction that occurs earlier than impairment of muscular contractility^4^. Several studies have reported that there may be deficiency of NGF in the diabetic bladder^25–29^, which is concordant with our finding of decreased levels of bladder NGF protein and mRNA in STZ-induced diabetic rats. Treatment with a hypoglycemic agent is reported to restore the normal expression of NGF^29^. It was speculated that impaired functions of Aδ- and C-fiber afferent pathways in diabetic bladder is related to the long-term decline of NGF expression in bladder and in L6 to S1 dorsal root ganglia^25^. In the present study, we examined the expressions of CGRP (a sensory nerve marker of Aδ-fiber) and substance P (a sensory nerve marker of C-fiber). Our results showed decreased expressions of sensory nerve markers which reflects that peripheral neuropathy induced by DM may cause the loss of neurotrophic support and dysfunction of detrusor contraction. Tong *et al*. reported the administration of ONO-2235 (China Chemical and Pharmaceutical, Taipei, Taiwan), an aldose reductase inhibitor, significantly improved the expressions of NGF protein and mRNA to normal levels in the diabetic bladder^30^. Treatment of ONO-2235 may also improve voided volume and residual volume in 9-week STZ-induced diabetic rats, which indicates diabetic bladder dysfunction may be associated with the down-regulation of NGF. However, the ONO-2235 treatment was found to be unable to reverse the structure changes such as bladder weight and capacity in the diabetic rats^30^. Our results showed that hAFSCs transplantation into bladder may significantly recover NGF protein and mRNA levels in the diabetic bladder and improve the voided volume, ICI and residual volume at week 4 and 12 after DM induction. A recent research^31^ demonstrated that NGF may be secreted by hAFSCs in the culture medium. It is possible the NGF secreted by hAFSC may help to enhance the neurotrophic effect after stem cell treatment in diabetic rats.

Besides the down-regulation of NGF in bladder and in L6 to S1 dorsal root ganglia, both motor and sensory functions of the bladder muscarinic mechanisms are suggested to be involved in the alternation of bladder function in STZ-induced diabetic rats^32,33^. The M2 and M3 receptors are present predominantly in rat bladders and play an important role to induce detrusor muscle contraction^34,35^. Previous studies demonstrated that the M2 and M3 protein and mRNA could be up-regulated in diabetic bladder at week 2 after DM induction^32,33,36,37^. It is postulated that the up-regulation of bladder muscarinic receptors can lead to overactivity of bladder at the early stage of DM^38^. Our results showed that the levels of M2 and M3 immunoreactivities and mRNA increased significantly in 4-week diabetic rats. Expression levels of M2 and M3 also showed an up-regulation at week 12 after DM induction but had no significant difference between diabetic and non-diabetic bladders. Pak *et al*. observed a significant increase in M2 function at 2 to 3 weeks and 20 to 24 weeks, but not at the intermediate time point (8 to 9 weeks) after DM induction^39^. In the present study, M2 and M3 protein and mRNA were recovered by the hAFSCs transplantation at week 4 after DM induction. However, hAFSCs transplantation did not alter the expressions of M2 and M3 in our 12-week diabetic rats. Tong *et al*. used ONO-2235 to treat STZ-induced diabetic rats and found a significant increase in the expressions of M2 protein and mRNA in the bladder tissue as compared to control rats^40^, which reflects diabetic bladder dysfunction may be related to the up-regulation of muscarinic receptors in the early phase of DM.

8OHdG is known a predominant form of free radical-induced oxidative DNA damage products and may act as a marker for DNA oxidative damage. Our data showed that the expression of 8OHdG increased significantly in the diabetic bladder at week 4 and 12 after DM induction. However, after hAFSCs transplantation, the bladder 8OHdG immunoreactivity reduced. Other studies in STZ-induced diabetic rats also found that oxidative stress could be implicated in the pathogenesis of diabetic complication^6,7^. It is likely that 8OHdG may be considered as one of the major markers of bladder dysfunction in diabetic rats.

Until now, few articles have reported the effects of stem cells to improve diabetic bladder dysfunction^8^. Zhang *et al*. found adipose stem cells may ameliorate diabetic bladder dysfunction in STZ-induced diabetic rats through predominantly paracrine effect and some cellular differentiation after stem cell transplantation^8^. Our study is the first to use hAFSCs for the treatment of diabetic bladder dysfunction. The hAFSCs can be obtained from amniotic fluid, grow easily in culture and appear phenotypically and genetically stable, which suggests that these stem cells may act as a novel source for cell transplantation therapy^10^. Further studies are needed to investigate the mechanisms of transplanted hAFSCs to improve diabetic bladder dysfunction.

The present study has some limitations. First, we used a single concentration of hAFSCs in this study. It is possible that higher concentration may have better effect on diabetic bladder dysfunction. Second, we examined the functional and morphological alterations of diabetic bladder at week 4 and 12 after DM induction, but it is possible to obtain better results if cystometry could be done at longer time points. Third, we did not directly examine the cellular differentiation after hAFSCs transplantation into bladder. Fourth, due to the complex nature of diabetic cystopathy, further study may be necessary to elucidate the major site of action and the long-term efficacy of hAFSC treatment. For example, whether hAFSC can inhibit bladder sensory nerve degeneration in chronic stages of DM or can decrease oxidative stress and inflammation.

In conclusion, our data support the inference that diabetic bladder dysfunction in STZ-induced diabetic rats can be improved by hAFSCs transplantation into bladder, which may be related to the recovery of bladder NGF and muscarinic receptors. It is possible that hAFSCs transplantation might be advantageous for patients with DM-related LUTS.

## Electronic supplementary material

Supplemental Figure 1-3
