# Supplementary material for: Improvement in bladder dysfunction after bladder transplantation of amniotic fluid stem cells in diabetic rats
Source: Sci Rep. 2018 Feb 1;8:2105. doi: 10.1038/s41598-018-20512-z (PMC5794746; doi:10.1038/s41598-018-20512-z)

Improvement in bladder dysfunction after bladder transplantation of amniotic fluid stem cells in diabetic rats

Ching-Chung Liang[1, 2], Sheng-Wen Steven Shaw[2, 3, 4], Yung-Hsin Huang[1],

Yi-Hao Lin[1, 2] & Tsong-Hai Lee [*, 2, 5]

[1]Female Urology Section, Department of Obstetrics and Gynecology, Chang Gung Memorial Hospital Linkou Medical Center, Taoyuan, Taiwan

[2]College of Medicine, Chang Gung University, Taoyuan, Taiwan

[3]Division of Obstetrics, Department of Obstetrics and Gynecology, Chang Gung Memorial Hospital Linkou Medical Center, Taoyuan, Taiwan

[4]Prenatal Cell and Gene Therapy Group, Institute for Women's Health, University College London, London, UK

[5]Stroke Center and Department of Neurology, Chang Gung Memorial Hospital Linkou Medical Center, Taoyuan, Taiwan

Supplemental Fig. 1. The negative control studies are performed without primary antibody and without primary and secondary antibodies for nerve growth factor (NGF), M2-muscarinic receptor (M2) and M3-muscarinic receptor (M3), calcitonin gene-related peptide (CGRP), substance P and 8-hydroxy-20-deoxyguanosine (8OHdG). No or mild immunoreactivity can be seen in the negative studies.

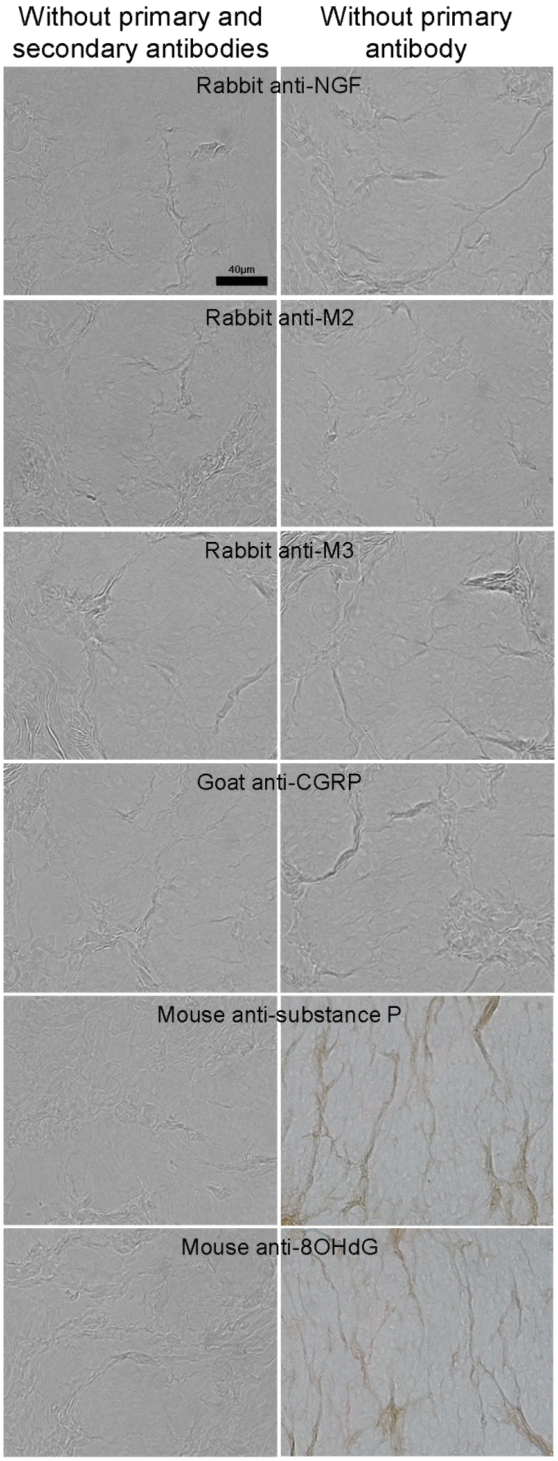

Supplemental Fig. 2. Temporal expressions of calcitonin gene-related peptide (CGRP) immunoreactivity in the bladder of control, STZ-induced diabetic rats (DM) and diabetic rats + hAFSCstransplantation (DM + hAFSCs). The immunoreactivity of CGRP can be seen in control rats (A), diabetic rats (B), and diabetic rats + hAFSCs transplantation (C) at 4 weeks, and in control rats (D), diabetic rats (E), and diabetic rats + hAFSCs transplantation (F) at 12 weeks. When compared to the controls, the expressions of CGRP in the diabetic rats are decreased at 4 and 12 weeks, but can be recovered to near the control level after hAFSCs transplantation. OD= optical density. Bar indicates 40 μm. **P* < 0.05 vs. control group. #*P* < 0.05 vs. diabetic rat. N = 10 in each time point.

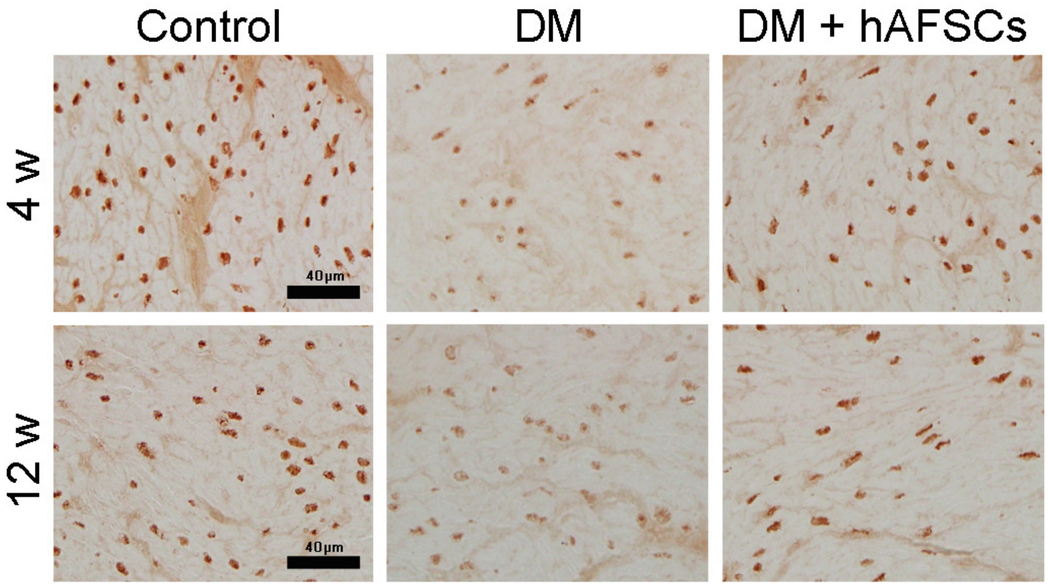

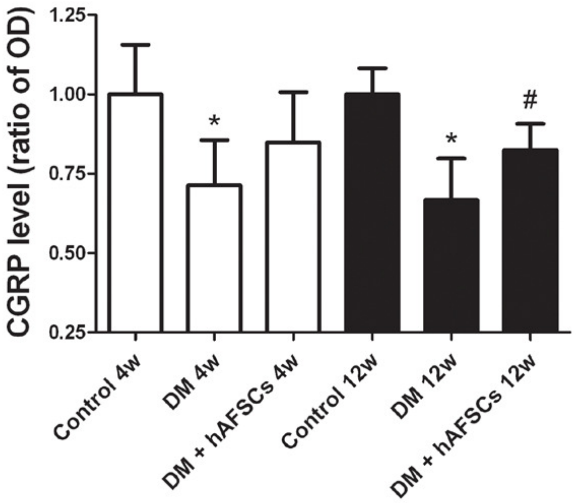

Supplemental Fig. 3. Temporal expressions of Substance P immunoreactivity in the bladder of control, STZ-induced diabetic rats (DM) and diabetic rats + hAFSCs transplantation (DM + hAFSCs). The immunoreactivity of Substance P can be seen in control rats (A), diabetic rats (B), and diabetic rats + hAFSCs transplantation (C) at 4 weeks, and in control rats (D), diabetic rats (E), and diabetic rats + hAFSCs transplantation (F) at 12 weeks. When compared to the controls, the expressions of substance P in the diabetic rats are decreased at 4 weeks, but can be recovered to near the control level after hAFSCs transplantation. There is no significant difference at 12 weeks. OD= optical density. Bar indicates 40 μm. **P* < 0.05 vs. control group. N = 10 in each time point.

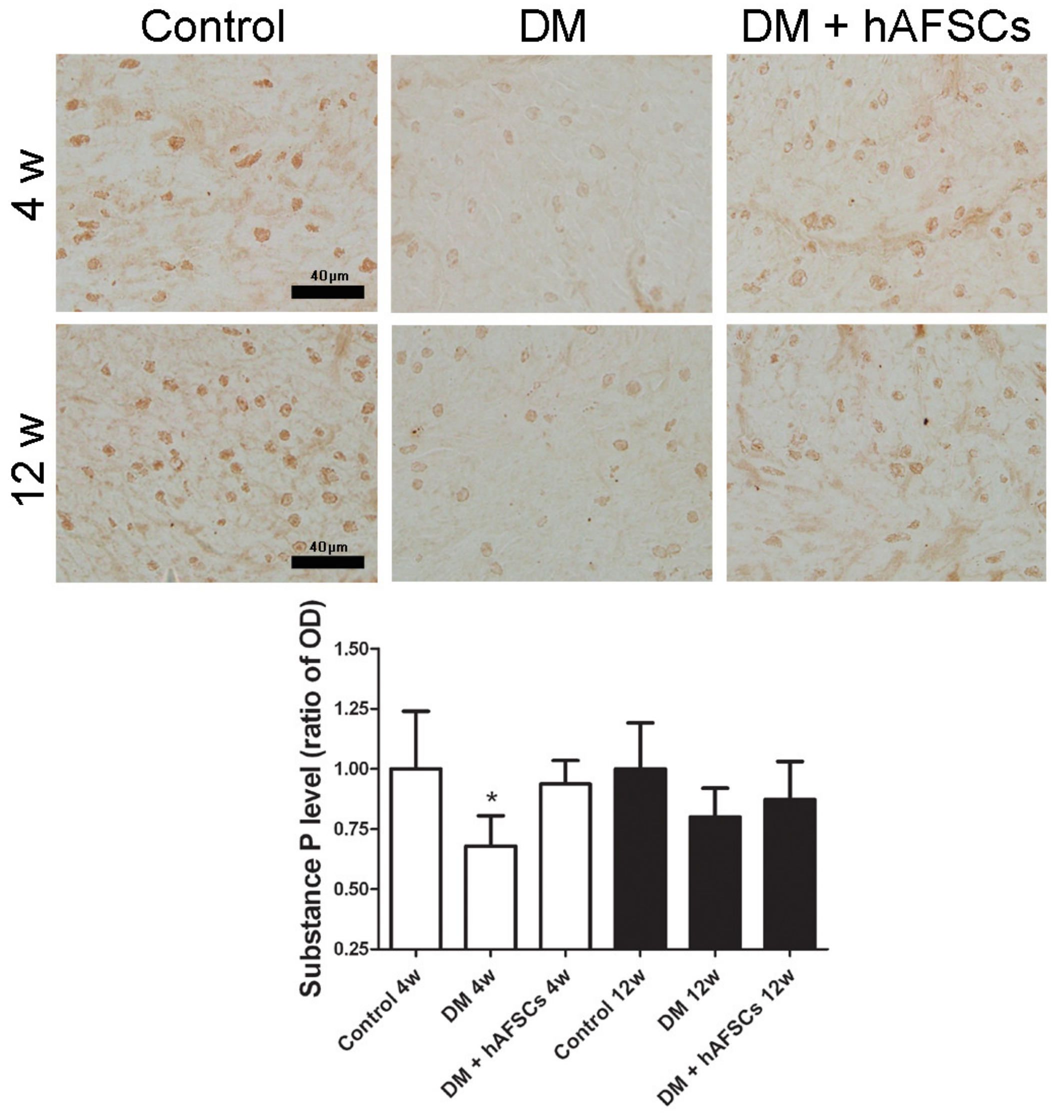

Supplement: Supplementary file 1 — Supplemental Figure 1-3 [file 41598_2018_20512_MOESM1_ESM.pdf]
